# Cell Cycle Regulation by Alternative Polyadenylation of CCND1

**DOI:** 10.1038/s41598-018-25141-0

**Published:** 2018-05-01

**Authors:** Qiong Wang, Guopei He, Mengmeng Hou, Liutao Chen, Shangwu Chen, Anlong Xu, Yonggui Fu

**Affiliations:** 1 State Key Laboratory for Biocontrol, Guangdong Province Key Laboratory of Pharmaceutical Functional Genes, Department of Biochemistry, School of Life Sciences, Sun Yat-sen University, Higher Education Mega Center, Guangzhou, 510006 P. R. China; 2 Beijing University of Chinese Medicine, 11 Bei San Huan Dong Road, Chao-yang District Beijing, 100029 P. R. China

## Abstract

Global shortening of 3′UTRs by alternative polyadenylation (APA) has been observed in cancer cells. However, the role of APA in cancer remains unknown. *CCND1* is a proto-oncogene that regulates progression through the G1-S phase of the cell cycle; moreover, it has been observed to be switching to proximal APA sites in cancer cells. To investigate the biological function of the APA of CCND1, we edited the weak poly(A) signal (PAS) of the proximal APA site to a canonical PAS using the CRISPR/Cas9 method, which can force the cells to use a proximal APA site. Cell cycle profiling and proliferation assays revealed that the proximal APA sites of CCND1 accelerated the cell cycle and promoted cell proliferation, but UTR-APA and CR-APA act via different molecular mechanisms. These results indicate that PAS editing with CRISPR/Cas9 provides a good method by which to study the biological function of APA.

## Introduction

Most human genes contain more than one poly (A) site, which leads to the prevalence of alternative polyadenylation (APA)^1^. There are two major types of APA: (1) untranslated region alternative polyadenylation (UTR-APA), which results in 3′UTR shortening without changing the coding region, and (2) coding region alternative polyadenylation (CR-PA), which produces different protein isoforms through the usage of poly(A) sites residing in an intron^2,3^. Global APA events have been reported to be associated with specific biological processes, including cancer development, metastasis, animal development, immune response, and neuronal activity^4–11^. It has been found that UTR-APA is related to mRNA stability and translation efficiency^6,10,12–14^; however, this does not directly explain the mechanism of APA in these biological processes. Distinct mRNA isoforms of *BDNF* produced by APA exhibit different subcellular localization in neurons^15^, and mouse mutants expressing *BDNF* with a truncated long 3′UTR were deficient in pruning and were characterized by enlarged dendritic spines^15^. By transducing cancer cells with shorter and longer isoforms of the *CCND2* and *IMP-1* genes, Mayr *et al*.^10^ found that the shorter isoforms of these two genes promote the cell cycle and increase colony formation. However, transducing exogenous genes cannot fully recapitulate the physiological effects of APA.

**Figure 1:**
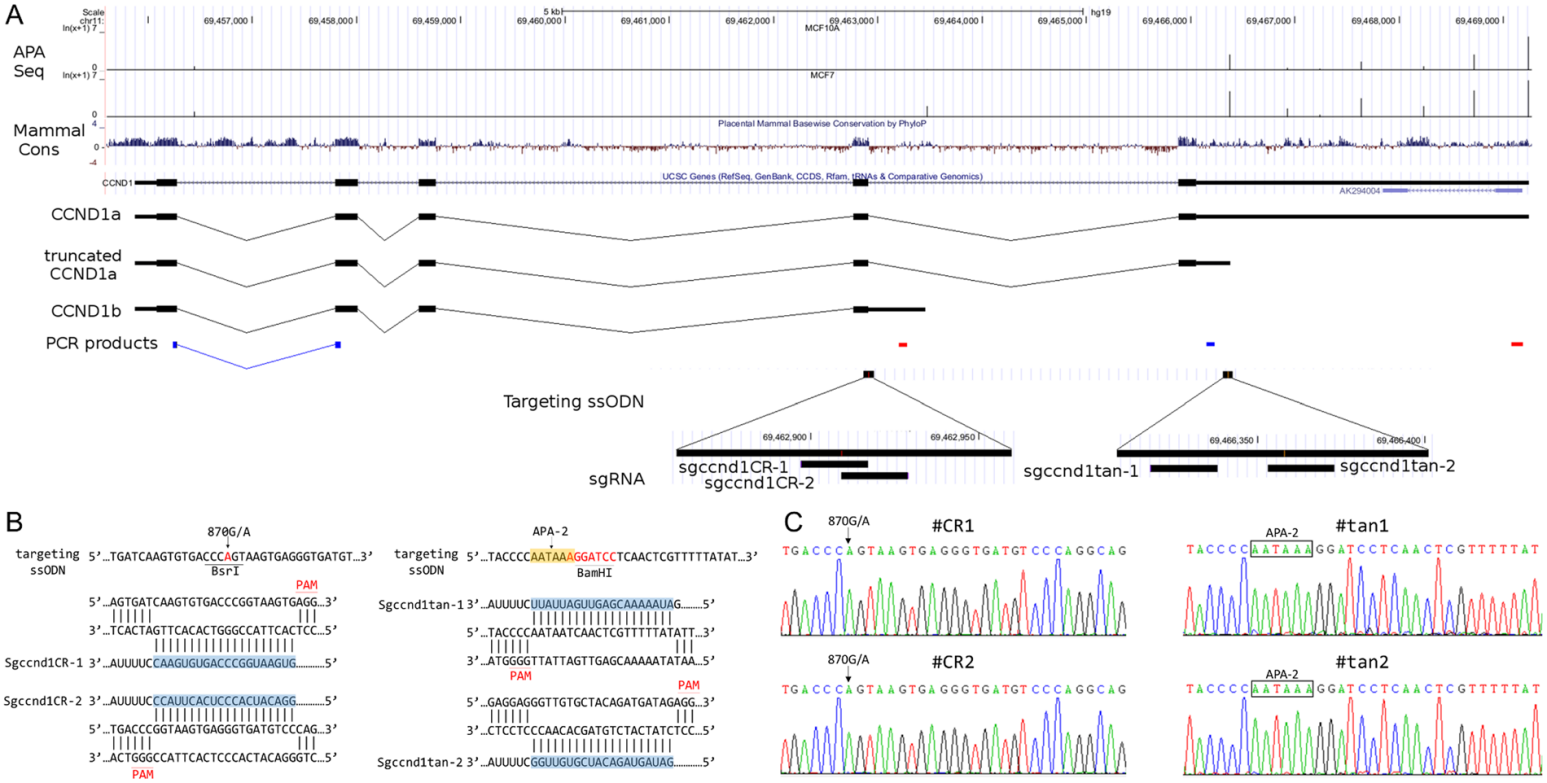
Alternative polyadenylation of *CCND1* and PAS editing with CRISPR/Cas9. (**A**) Upper panel: APA switching in breast cancer cell lines. MCF10A is a human normal mammary epithelial cell line; MCF7 is a human breast cancer cell line. Lower panel: Schematic representation of the *CCND1* locus, APA sites, mRNA isoforms, sgRNA and ssODN. qRT-PCR products used to quantify usage of the APA sites are also shown; the first two correspond to the APA-1 site (CR-APA) and the last two are for the APA-2 site (UTR-APA). Blue represents the common region and red represents the extended region. (**B**) Sequences of the single-stranded oligonucleotides (ssODN) and sgRNAs used to target the locus. Two sgRNAs were designed for each APA site. Left panel (870 G/A for APA-1): “G” at position 870 is replaced by “A”, which introduces a BsrI site “CCCAGT”; Right panel (APA-2): “AGGATCC” was inserted following “AATAA” at position 304 bp upstream of the stop codon, introducing a canonical PAS “AATAAA” site and a BamHI site. (**C**) Sequencing validation of the mutated cell lines. #CR1 and #CR2 clones were mutated to use the APA-1 site with sgccnd1CR-1 and sgccnd1CR-2, respectively. #tan1 and #tan2 clones were mutated to use the APA-2 site with sgccnd1tan-1 and sgccnd1tan-2, respectively.

Cyclin D1 (*CCND1*), which is frequently aberrant in human cancers^16,17^, plays a critical role in promoting the G1–S transition of the cell cycle in many cell types^18,19^. *CCND1* is subject to both UTR-APA and CR-APA (Fig. 1A). In tumor cell lines and cancer patients, two major isoforms of *CCND1* have been identified: CCND1a, which contains exons 1–5, and CCND1b, which ends with a longer exon 4 and is created by CR-APA using poly(A) sites within intron 4^20–23^. Previous studies have found that the expression of CCND1b is tightly correlated with an 870 G/A polymorphism at the last base of exon 4 (position 870, codon 241). Furthermore, two mantel cell lymphoma patients harbor mutations in exon 5 (position 304 bp downstream of the stop codon), that produce a novel poly(A) signal (PAS: AAUAAA) and an isoform of CCND1a mRNA with a shorter 3′UTR (truncated CCND1a)^20^. Using the 3′ end sequencing technologies SAPAS and IVT-SAPAS, we observed expression of truncated CCND1a, albeit without a PAS, at this APA site in the breast cancer cell lines MCF7 and MB231 and in the mammary epithelial cell line MCF10A^24,25^. We also found that switching to the truncated isoform was more common in the breast cancer cell lines compared to MCF10A (Fig. 1A).

To investigate the effects of APA on endogenously expressed *CCND1*, we performed PAS editing with CRISPR/Cas9 in the 293T cell line to express truncated CCND1a and CCND1b. We found that proximal APA elevated the expression levels of both CCND1 protein and mRNA. Moreover, the truncated CCND1a isoform did indeed promote cell proliferation and accelerate cell cycle progression. Thus, we successfully studied the biological function of APA of *CCND1* through PAS editing with the CRISPR/Cas9 system, a method that can be used for future studies of APA function.

## Results

### PAS editing with CRISPR/Cas9

To endogenously express CCDN1b and truncated CCND1a, we performed gene editing for APA-1 and APA-2 using CRISPR/Cas9 in the 293T cell line. Two sgRNA sequences for each isoform (truncated CCND1a: sgccnd1tan-1 and sgccnd1tan-2, CCND1b: sgccnd1CR-1 and sgccnd1CR-2; Fig. 1A,B were designed at http://crispr.mit.edu/, and cloned into the pX459 plasmid (Addgene), which expresses human codon-optimized Cas9. The donor sequences of single-stranded oligo–nucleotides (ssODN) were synthesized as follows (Fig. 1A,B): 1) for truncated CCND1a, “AGGATCC” was inserted following “AATAA” at position 304 bp downstream of the stop codon, thereby introducing a canonical PAS and a BamHI site into the 3′UTR; 2) for CCND1b, “G” at position 870 was replaced by “A”, thereby introducing a BsrI site. A surrogate RFP-GFP reporter system^26^ was also used to screen for cells positive for the Cas9 modification.

Cas9-sgRNA, the RFP-GFP reporter plasmid, and ssODNs were co-transfected into HEK293T cells, and single GFP-positive cells were sorted into a 96-well plate by fluorescence-activated cell sorting (FACS). We then screened individual clonal cell populations using PCR-RFLP to identify mutants. In total, 3 and 9 homozygous mutants for truncated CCND1a and CCND1b, respectively, were found and confirmed by Sanger sequencing (Fig. 1D). The mutated cell lines (#CR1 and #CR2 for APA-1, #tan1 and #tan2 for APA-2) were chosen for functional analysis.

**Figure 2:**
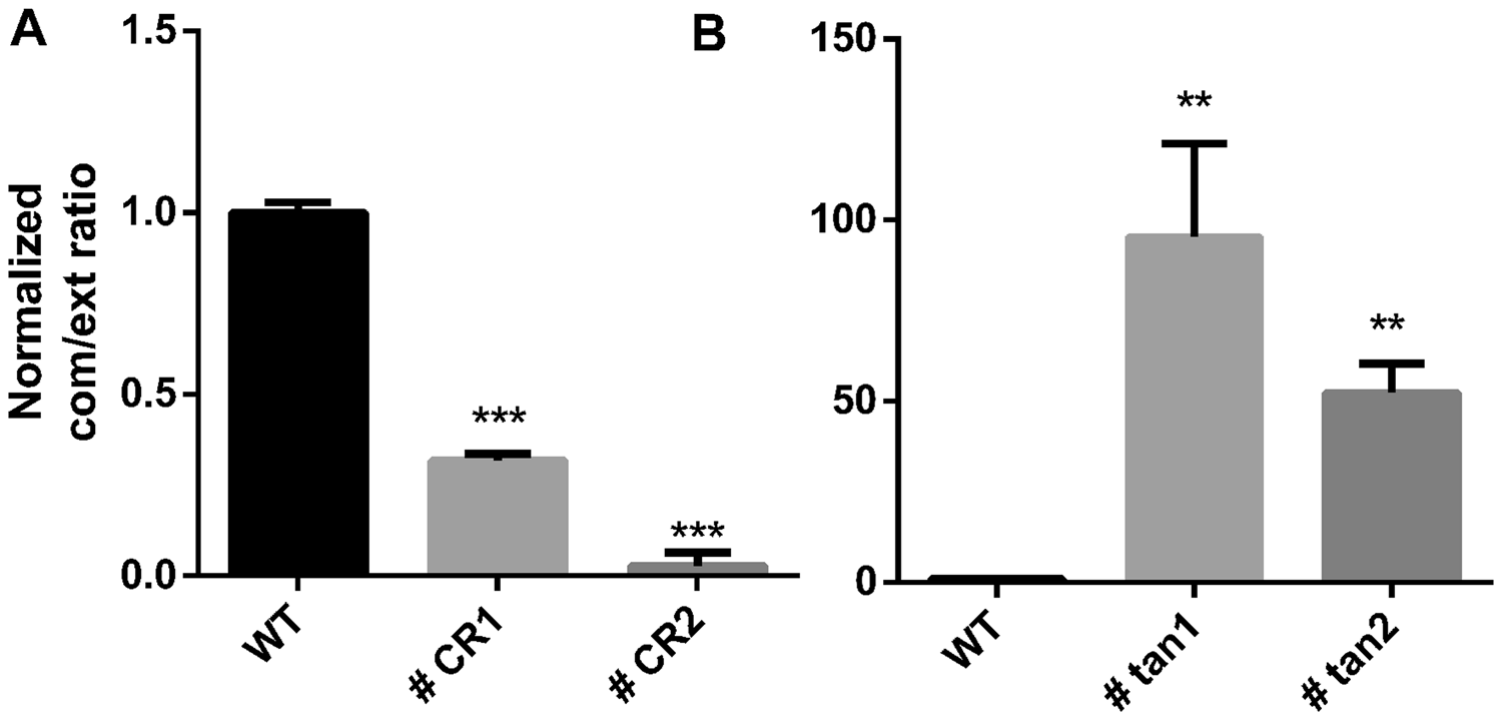
qRT-PCR validation of APA switching in mutated cell lines. qRT-PCR was performed with the primers shown in Fig. 1 and Supplementary Table 1. The ratio of common/extended products in mutated cell lines was normalized to that of the wild type cell line. The #CR1 and #CR2 clones were mutated to use the APA-1 site with sgccnd1CR-1 and sgccnd1-CR2, respectively, and the #tan1 and #tan2 clones were mutated to use the APA-2 site with sgccnd1tan-1 and sgccnd1tan-2, respectively. Data are represented as the mean +/− SEM (n = 3); Student’s t-test: *P < 0.05, **P < 0.01, ***P < 0.001.

We first performed 3′-RACE for CCND1b in the mutated cell lines. We found that CCND1b was significantly expressed in the cell lines #CR1 and #CR2 but was not expressed in the other cell lines (Supplementary Fig. 1). Sanger sequencing of the PCR product showed that the 3′ end was the same as our previous IVT-SAPAS results (Supplementary Fig. 1), suggesting that editing of the site 870 G/A is sufficient to activate use of the APA-1 site. To identify APA switching in the mutated cell lines, we measured the common/extended expression ratio using qRT-PCR. For the APA-1 site, two pairs of primers were designed to amplify the exon 1–2 junction (common region) and intron 4 (extended region); for the APA-2 site, two pairs of primers located within the common and extended 3′UTR at the last exon were designed (Fig. 1A). In the #CR1 and #CR2 cell lines, the common/extended ratio normalized to that of the wild type cell line for the APA-1 site was significantly less than one, indicating successful APA-1 site switching in these cell lines. In the #tan1 and #tan2 mutated cell lines, the common/extended ratio for the APA-2 site was significantly greater than one, revealing APA-2 site switching (Fig. 2). Next, we measured use of the APA-1 site in the cell lines #tan1 and #tan2 and of the APA-2 site in the cell lines #CR1 and #CR2 (Supplementary Fig. 2). We detected a weakly higher common/extended ratio for the APA-1 site in #tan2 but not in #tan1 and no change in the common/extended ratio for the APA-2 site in #CR1 and CR2. These results suggest successful APA switching by PAS editing in these cell lines.

### CCND1b and truncated CCND1a accelerate cell cycle progression

**Figure 3:**
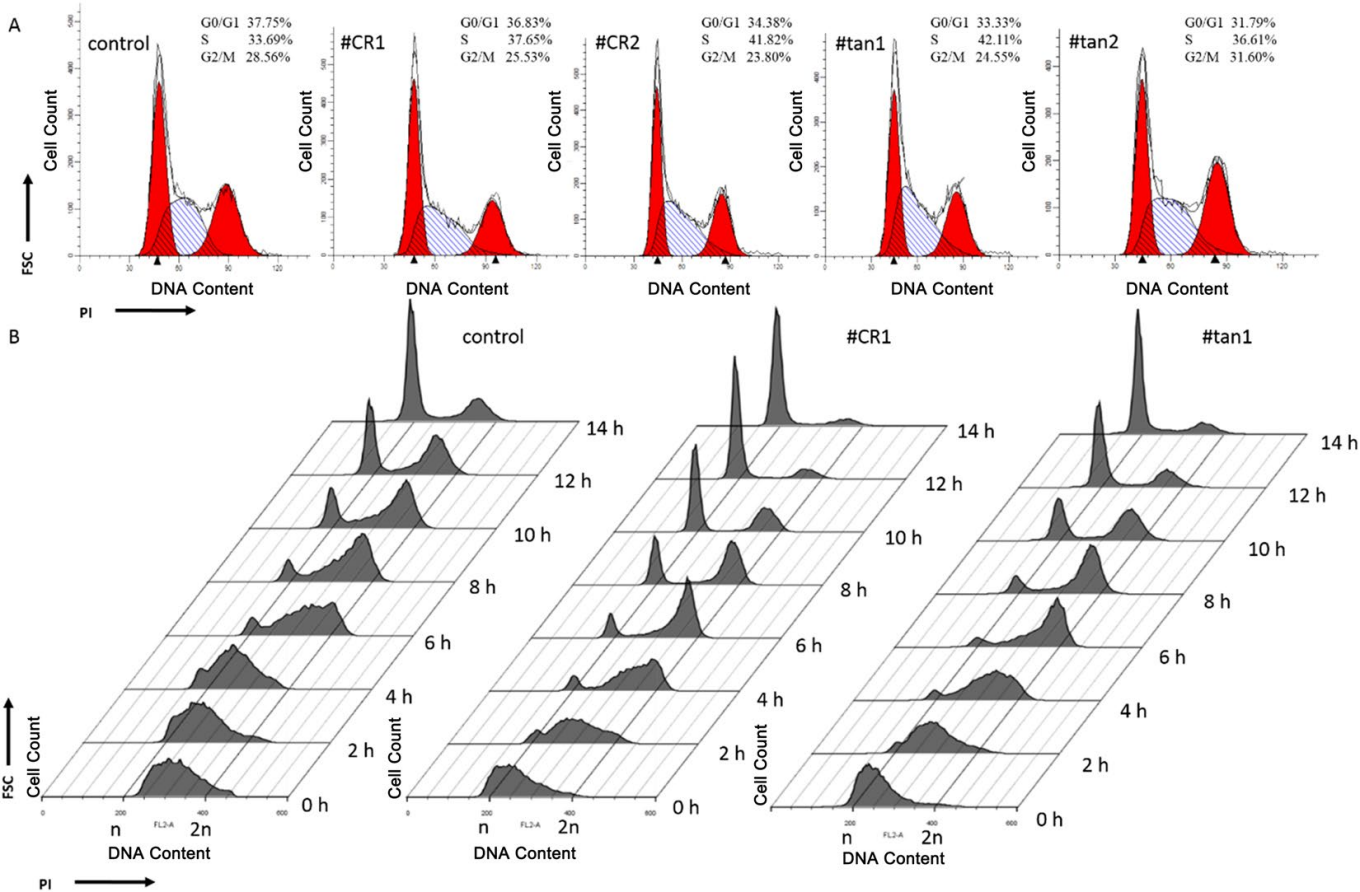
APA switching of *CCND1* accelerates cell cycle progression. (**A**) Cell cycle analysis by DNA content. Synchronized cells were stained with propidium iodide after 6 hours in culture, and percentages of the cell population in the different phases of the cell cycle were determined by flow cytometry. (**B**) Dynamic cell cycle profiles. Cells were arrested in the G1/S phase with a double thymidine block and were released at several time points. Cell cycle profiles were obtained by flow cytometry after propidium iodide (PI) staining.

*CCND1* plays a critical role in the cell cycle and tumorigenesis. To analyze the effects of *CCND1* APA switching of *CCND1* on the cell cycle, propidium iodide (PI) staining was used to distinguish the percentage of the cellular population that is in each phase of the cell cycle (G0/G1, S, and G2/M). #CR1 and #CR2 clonal cell populations exhibited a slight decrease in the percentage of the cellular population in the G0/G1 phase and an increase in the percentage of the cellular population in the S phase, compared to control cells (Fig. 3A). Moreover, the percentage of the cellular population in the S phase in #tan1 and #tan2 clonal cell populations was greater than observed for control cells as well as #CR1 and #CR2 clonal cell populations (Fig. 3A). Thus, both UTR-APA and CR-APA of *CCND1* may play an important role in cell cycle progression. To further study the dynamics of the cell cycle profile following *CCND1* APA switching, 293T cells were arrested at the G1/S boundary using a double thymidine block followed by replacement with fresh media (release media) to allow synchronized cells to progress through the cell cycle. As shown in Fig. 3B, in the release media condition, most of the control cells progressed into the G2/M phase at 2–4 h post-release and began to shift to the next G1 phase from the G2/M phase at 4–6 h. Most cells were located in the G1 phase of the next cycle by 14 h post-release. In contrast, #CR1 and #tan1 cells showed an accelerated cell cycle progression acceleration compared with control cells. Cell populations progressed into the G2/M phase at 0–2 h post-release. Furthermore, #CR1 and #tan1 cells entered the next G1 phase at 2–4 h, and two distinct G0/G1 and G2/M peaks appeared. Furthermore, most #CR1 and #tan1 cells were in the G1 phase of the next cycle by 12 h. These results suggest that the two *CCND1* mutations accelerate cell cycle progression.

### CCND1b and truncated CCND1a can promote cell proliferation

Because *CCND1* has been associated with stimulating proliferative responses in cell culture^27,28^, we investigated the effect of *CCND1* APA switching on cell proliferation with a BrdU incorporation assay. BrdU, an analogue of the DNA precursor thymidine, can be incorporated into newly synthesized DNA during S phase of the cell cycle and is usually used to determine the percentage of proliferating cells.

**Figure 4:**
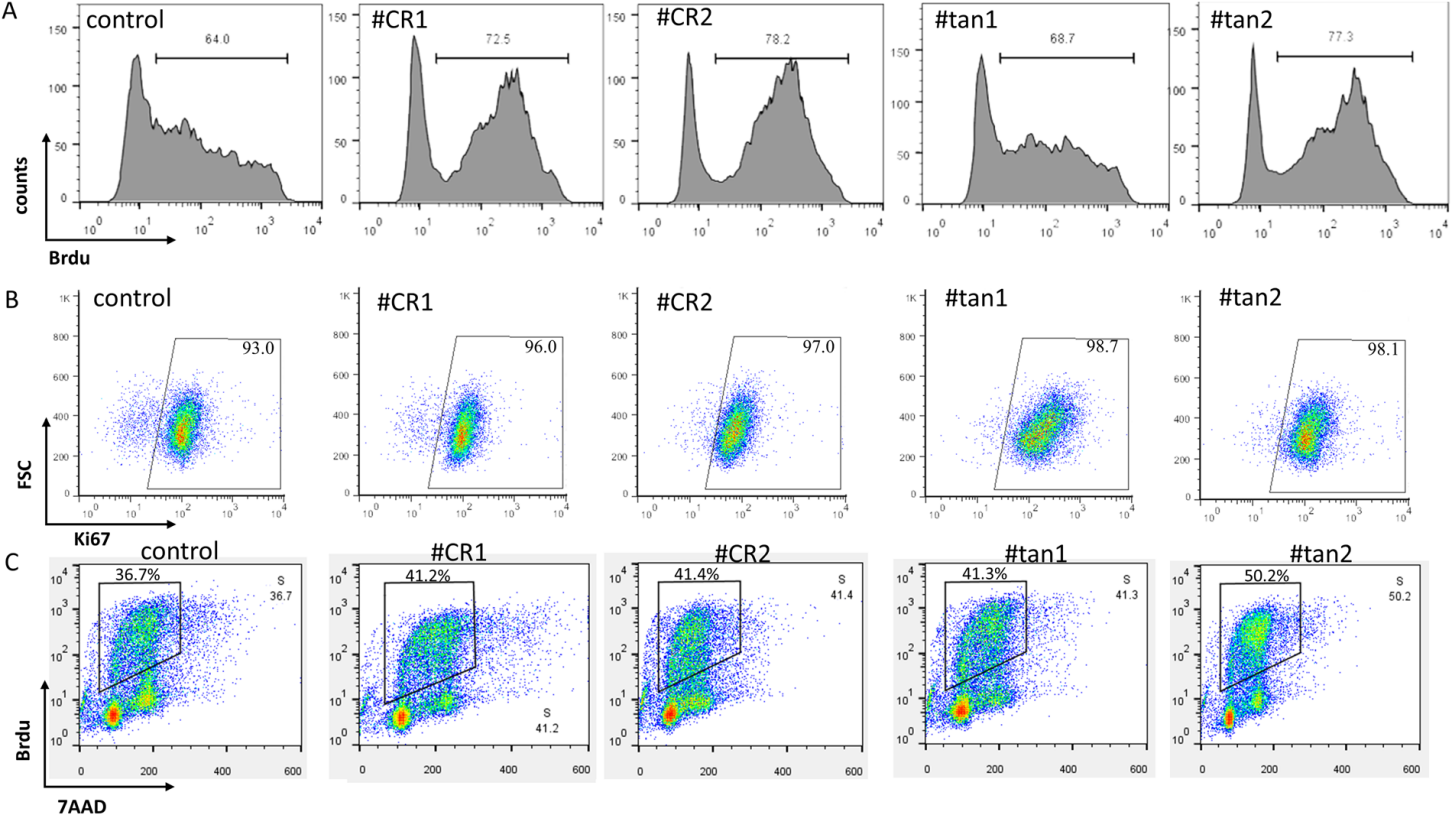
APA switching of *CCND1* promotes cell proliferation. (**A**) Cell proliferation was assessed by BrdU assay with flow cytometry. The proportions of proliferative cells are shown. (**B**) Proportion of Ki67 positive cells in mutant cells. Gates were set using isotype control antibodies. (**C**) Quantification of cell-incorporated BrdU and total DNA content (7-AAD).

The cell lines #CR1 and #CR2 were characterized by a higher percentage of BrdU-positive cells than control cells (72.5% and 78.2% vs. 64.0%), and the cell lines #tan1 and #tan2 also showed higher BrdU incorporation (68.7% and 77.3%, respectively) (Fig. 4A). Ki67 is expressed during all active phases of cell division but is absent in quiescent cells and during DNA repair^29^. We performed a Ki67 assay to determine the role of *CCND1* APA switching in cell proliferation. In control cells, the percentage of Ki67-negative cells among all mutated cell lines was lower than that of the wild type line, suggesting the presence of fewer quiescent cells in the mutated cell lines (Fig. 4B). To further validate these results, we evaluated the percentage of S phase cells by BrdU and 7-AAD double staining. Similarly, a higher proportion of cells in S phase was observed in the mutated cell lines compared to wild type cells (Fig. 4C). Together, these results confirm that a preference for proximal PAS usage at the *CCND1* locus can indeed promote cell proliferation.

### Distinct molecular mechanisms of truncated CCND1a and CCND1b

**Figure 5:**
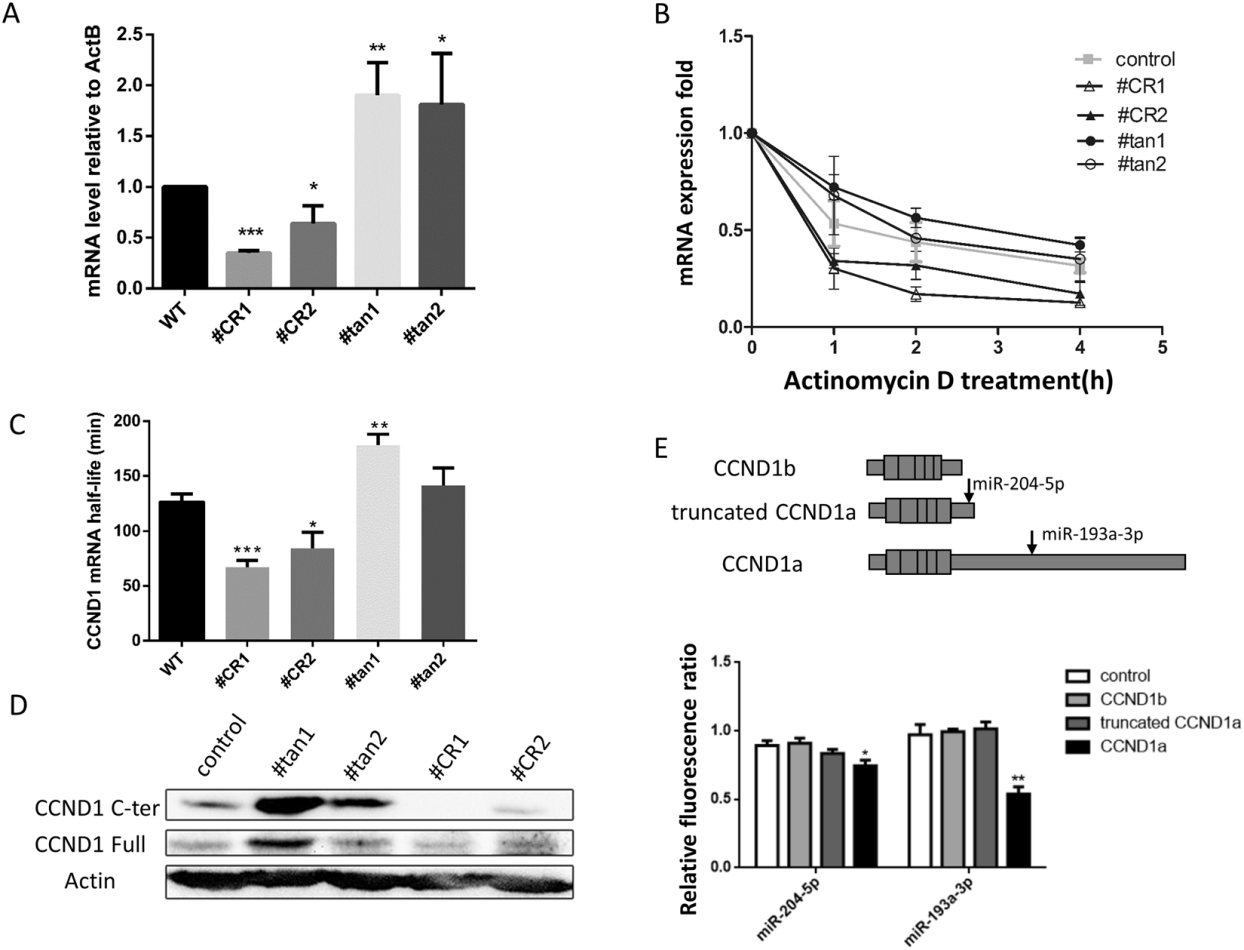
Effects of APA on mRNA expression level and stability. (**A**) *CCND1* mRNA expression levels were measured by qRT-PCR. Student’s t-test: *P < 0.05, **P < 0.01, ***P < 0.001. (**B**) The mRNA decay rate was measured by qRT-PCR. The relative expression levels of *CCND1* transcripts at the APA-1 site in mutated and wild type cells were analyzed at 0 h, 1 h, 2 h and 4 h after inhibition of transcription with 10 µg/mL actinomycin D. Data at each time point are representative of three independent experiments and are normalized to ACTB expression. (**C**) mRNA half-life. Student’s t-test: *P < 0.1, **P < 0.05, ***P < 0.01. (**D**) Western blot detection of CCND1 protein. The gels were run under the same experimental conditions. Cropped gels/blots are presented. (Full-length gels/blots are shown in Supplementary Fig. 3 with indicated cropping lines). (**E**) Repression of different CCND1 3′UTR isoforms by miRNAs. Upper panel: Schematic representation of predicted miRNA binding sites. Lower panel: Repression of *CCND1* mRNA isoforms with miRNA, as measured with dual luciferase assay. 293T cells were co-transfected with miRNAs and psiCheck-2 plasmids carrying different *CCND1* 3′UTRs, and the ratio of Renilla and Firefly luciferase activities was determined. The data were normalized to measurements of cells co-transfected with empty psiCheck 2 negative control miRNA. Student’s t-test: *P < 0.1, **P < 0.05, ***P < 0.01.

To understand the mechanism of the *CCND1* APA in regulating the cell cycle and proliferation, we first determined the level of mRNA expression in these cell lines using common primers for APA-1 site designed to amplify the exon 1–2 junction. We found that CR-APA (#CR1 and #CR2) and UTR-APA (#tan1 and #tan2) exhibited opposite effects on the expression level of *CCND1* mRNA. The #CR1 and #CR2 cell lines exhibited significantly reduced levels of *CCND1* mRNA compared to the wild type cell line, whereas significantly higher mRNA expression was observed in #tan1 and #tan2 cells (Fig. 5A).

It is known that the 3′UTR plays an important role in mRNA stability; therefore, we measured the stability of *CCND1* mRNA in these cell lines. We treated the cells with Actinomycin D to block transcription, collected total RNA at time points of 0, 1, 2 and 4 hours after the treatment, and measured *CCND1* mRNA expression level in the samples by qRT-PCR. CCND1b mRNA in cell lines #CR1 and #CR2 decayed significantly faster than in wild type cells (Fig. 5B). Furthermore, the mRNA half-life in #CR1 (67.0 min) and #CR2 (84.3 min) cells was significantly shorter than in wild type cells (126.5 min) (p < 0.01 and p < 0.1, respectively) (Fig. 5C). The reduced stability of CCDN1b is consistent with the lower mRNA expression level in the #CR1 and #CR2 cell lines. However, truncated CCND1a in cell lines #tan1 and #tan2 was more stable (p < 0.05) (Fig. 5B,C), with mRNA half-lives of 178.3 and 141.2 min, respectively.

We also measured CCND1 protein level by Western blot. Higher CCND1 protein levels were observed in cell lines #tan1 and #tan2 using either an anti-CCND1 C-terminus antibody or an antibody against full-length CCND1 (Fig. 5D), consistent with the higher mRNA expression levels observed in these cells. The protein levels detected were slightly decreased when using the full-length antibody in cell lines #CR1 and #CR2 and could not be detected using the C-terminus antibody due to loss of the amino acid residues at the carboxyl terminus (Fig. 5D).

miRNA plays an important role in altering mRNA and protein levels. The potential binding sites of miRNAs targeting different CCND1 3′UTR were predicted using TargetScan software. The CCND1a 3′UTR common region contains an miR-204-5p binding site, while the extended 3′UTR region contains an miR-193a-3p binding site. We used dual luciferase reporter assays to study the effects of miRNAs. CCND1b 3′UTR and CCND1a short and long 3′UTRs were cloned into the psiCHECK-2 vector downstream of Renilla luciferase reporter gene. The dual-luciferase reporter vectors were co-transfected with miRNAs into HEK293T cells. We found that overexpression of miR-193a-3p significantly inhibited the expression of CCND1a but not of truncated CCND1a. Furthermore, miR-204-5p slightly decreased the expression of CCND1a (Fig. 5E). The reporter gene assay indicates that miRNAs may be linked to differences in mRNA stability and protein level between CCND1a and truncated CCND1a.

These results suggest that switching to the *CCND1* APA-2 site can elevate mRNA and protein expression levels, whereas utilizing the *CCND1* APA-1 site decreases CCND1a expression but increases CCND1b expression level. Both of the sites result in the acceleration of the cell cycle and promote cell proliferation. However, the reduced stability and expression of CCND1b suggests that the CCND1b protein differs in function from the CCND1a protein due to the loss of amino acid residues at its carboxyl terminus (see Discussion).

## Discussion

With the development of high throughput technologies (e.g., microarray and next generation sequencing), UTR-APA has been linked to cancer, embryonic development, tissue specificity, immune response, and neuronal activity. UTR-APA can lead to changes in 3′UTR length, which can in turn impact the stability, translation efficiency and subcellular localization of mRNA. UTR-APA can also regulate protein transport^30^ by altering the interaction of RNA binding proteins with the 3′UTR. Through these functions, UTR-APA plays a role in gene regulatory networks and contributes to various biological phenotypes. Although many studies have investigated the molecular function of UTR-APA, we lack direct observations of the effect of UTR-APA on phenotype. The transduction of plasmids expressing different mRNA isoforms provides a way to measure phenotypic changes resulting from UTR-APA. However, it also alters the expression level due to use of a different promoter or the chromatin status compared to endogenous genes.

Making mutations to endogenous genes that force the use of one of the APA sites to investigate the biological function of UTR-APA is a preferred method. The BDNF gene in mice has two UTR-APA sites; the *BDNF* mRNA with a shorter 3′UTR has localization that is restricted to the soma of neurons while the longer isoform can also be transported into the dendrites of neurons. In a mouse mutant with a new APA site between the two UTR-APA sites of *BDNF*, there is little *BDNF* mRNA and protein in dendrites and the mutant exhibits dysmorphogenesis of dendritic spines^15^.

The CRISPR/Cas9 system is a useful tool to edit the genome precisely. In this study, we successfully mutated the 293T cell line to use APA-1 and APA-2 sites with CRISPR/Cas9. With this endogenously expressed CCND1b and truncated CCND1a, we investigated the molecular effect on mRNA stability in addition to the cell cycle and proliferation rate. We found that both of APA-1 and APA-2 sites can accelerate the cell cycle and promote cell proliferation. This phenomenon is consistent with observations from Mantle cell lymphoma patients^20^. Higher mRNA and protein expression levels were observed in the APA-2 mutated cell lines, suggesting that APA-2 site can drive the cell cycle and cell proliferation by elevating CCND1 protein levels. In cell lines with a mutated APA-1 site, we found increased instability and a lower expression level of CCND1b mRNA; moreover, previous reports also found that the CCND1b protein showed similar stability to CCND1a protein^28^. Therefore, the mechanism that leads to an accelerated cell cycle and higher proliferation rate should be different from APA-2 site. CCND1b protein is constitutively localized to the nucleus while CCND1a protein is transported into cytoplasm in S-phase^28^. This localization difference may have contributed to the effect of CCND1b on cell proliferation.

Furthermore, we observed an increased mRNA expression level in the cell lines #tan1 and #tan2, which have been mutated to the APA-2 site with a strong PAS. We know that mRNA expression level is positively correlated with the rate of mRNA production and half-life. Although truncated CCND1a was found to have slightly increased mRNA stability, this change may not be enough to explain the expression difference. This indicates that using the APA-2 site can affect the mRNA expression level by affecting the transcription process. It has been reported that gene looping is formed through the juxtaposition of promoter and terminator regions by 3′ end processing factors, such as Ssu72 and CF1A in yeast^31,32^. Mapendano *et al*.^33^ found that a poly(A) site mutation of the *β-globin* gene can decrease transcriptional initiation of this gene and that 3′ end formation can stimulate transcription initiation. All of the above suggest that APA may also affect transcriptional initiation and elongation rate in addition to the known effects of mRNA stability, localization and translation efficiency. This should be further investigated by integrating chromosome conformation capture and APA analysis.

## Materials and Methods

### Cell culture and transfection

The human embryonic kidney cell line HEK293T was cultured in Dulbecco’s modified Eagle’s Medium (DMEM; Gibco) supplemented with 10% fetal bovine serum (FBS; Gibco) and 1% penicillin-streptomycin (Gibco) at 37 °C with 5% CO_2_ incubation. Cells were transfected using Lipofectamine 2000 transfection reagent (Invitrogen) according to the manufacturer’s instructions.

### sgRNA vectors construction and donor DNA preparation

The human codon-optimized Cas9 and sgRNA expression plasmid px459 was obtained from Addgene. For construction of sgRNA vectors, the sgRNAs were synthesized, annealed and ligated to the pX459 plasmid, which was digested with BbsI (New England Biolabs). Single-stranded oligo–nucleotides (ssODN) were synthesized by Beijing Genomics Institute. The sequences of sgRNAs and ssODN are described in Supplementary Table 1.

### RFP-GFP reporters

The empty RFP-GFP reporter plasmid pRGS was constructed as described previously^26^. For construction of indicated reporter vectors, oligonucleotides that contained *CCND1* target sites were synthesized and annealed *in vitro*. The annealed oligonucleotides were ligated into pRGS digested with EcoR1 and BamH1. The sequence of the reporter that contained *CCND1* target sites is described in Supplementary Table 1.

### Fluorescence-activated cell sorting to enrich Cas9 positive cells

For flow cytometry sorting, HEK293T cells seeded into 6-well plates were transfected with 2 μg of sgRNA vector, 2 μg of RFP-GFP reporter and donor DNA (2 μl ssODN [10 μM]). Three days after transfection, cells were trypsinized and prepared in single-cell suspensions with PBS/2% FBS buffer and sorted with a FACS Aria II machine (BD Bioscience). Untransfected cells and cells transfected with GFP alone were used as controls. For the bulk sorting, 2 × 10^5^ GFP-positive cells were sorted into 5-ml Falcon tubes with complete medium. Cells were then centrifuged and used for extraction of genomic DNA. For single-cell cloning, single GFP-expressing cells were sorted into 96-well plates with 200 μl complete medium per well. These plates were incubated at 37 °C, 5% CO_2_. Cells were cultured until confluency and duplicated for genotyping PCR.

### RFLP assay for detection of homologous recombination

Genomic DNA was extracted using the QuickExtract DNA extraction kit (Epicentre) following the manufacturer’s instructions. The target genomic region was PCR amplified using primers outside the homology arms of the homologous recombination (HR) template listed in Supplementary Table 1. PCR products were separated on a 1% agarose gel and extracted with MinElute GelExtraction Kit (Qiagen). Purified products were digested with HindIII or BsrI (Fermentas) and analyzed on DNA-PAGE.

### Real-time RT-PCR

Total RNA was isolated from cells with TRIzol reagent (Invitrogen, Carlsbad, CA) according to the manufacturer’s instructions. After digestion DNA with Ambion TURBO DNA-free^TM^ Kit, 500 ng of total RNA was reverse transcribed to synthesis cDNA with random hexamer primers and oligo (dT) primers using ReverTra Ace qRT-PCR RT Master Mix with gDNA Remover (Toyobo, Japan). A total of 5 μl of cDNA (1:10 dilution) was amplified using SYBR®Green Realtime PCR Master Mix (Toyobo, Japan) and performed on a LightCycler 480 (Roche). The relative expressive level of the target genes was calculated using the ΔΔCt method with control β-actin. Three biological replicates were performed. Primers for qPCR analyses are listed in Supplementary Table 1.

### mRNA half-life analysis

For *CCND1* mRNA half-life analysis, HEK293T single clonal cell lines were treated with 10 μg/mL actinomycin D (Sigma) to inhibit transcription and were harvested at the indicated time points. qRT-PCR to measure the decay in RNA abundance was carried out as described above with the common primers located in *CCND1* exon 1 and exon 2 (listed in Supplementary Table 1). Next, linear regression was performed between log values of the expression levels and the time points and half-life was calculated.

### Western blot analysis

Whole cells were lysed in RIPA lysis buffer at 4 °C for 30 min. Proteins (20 µg of protein/lane) were separated by SDS-PAGE and transferred to nitrocellulose membranes using a semi-dry transfer system (Bio-Rad). The membrane was blocked in 5% non-fat milk and incubated with anti-CCND1 C-terminus antibody (Cell Signaling), anti-CCND1 full length antibody (Thermo Fisher Scientific) or anti-Actb antibody (Proteintech) overnight at 4 °C. Then, the membrane was washed with TBS-T and incubated with corresponding horseradish-peroxidase-labelled secondary antibodies (Cell signaling) for 1 h. Protein signals were detected by supersignal west femto maximum sensitivity substrate (Thermo scientific).

### Cell cycle analysis by DNA content

Cells were synchronized by starvation in DMEM supplemented with 1% FBS for 72 h and then released into the cell cycle by stimulation with 10% FBS for 6 h before cell cycle analysis. Cells were harvested with trypsinization and fixed in 70% ethanol overnight at −20 °C. After washing with phosphate-buffered saline (PBS), the cells were incubated with 0.2 mg/ml DNase-free RNase A (Sigma), 20 μg/ml propidium iodide (Sigma) and 0.1% v/v Triton X-100 (Sigma) in PBS for 30 min on ice in the dark. Cell data were collected with a FACS Calibur flow cytometer (BD Biosciences) and analyzed by Modfit software.

### Cell cycle profile analysis by double thymidine block synchronization

Cells were grown to 50% confluency in complete medium. Thymidine (2 mM, Sigma) was added and the cells were incubated for 18 h, washed three times with PBS, and released into thymidine-free complete medium for 9 h. Thymidine (2 mM) was then added for an additional 15 h. Prior to cell cycle profile analysis, the cells were washed three times with PBS and cultured in complete medium without thymidine. The cells were collected at different time points (0, 2, 4, 6, 8, 10 and 12 h) after double thymidine block and were analyzed by PI staining as described above.

### BrdU incorporation assay

A 5-bromo-2 deoxyuridine (BrdU) incorporation assay was conducted to monitor DNA replication using a FITC BrdU Flow Kit (BD Bioscience) according to the manufacturer’s instructions. Briefly, cells were synchronized by starvation in DMEM supplemented with 1% FBS for 72 h before treatment with 10% FBS and 100 µM BrdU for 6 hours. The cells were fixed and permeabilized with BD Cytofix/Cytoperm Buffer, BD Buffer Plus, and BD Cytofix/Cytoperm Buffer in sequence. Then, cells were treated with DNase before incubation with an anti-BrdU-FITC antibody with or without PI. Cell data were collected with a FACS Calibur flow cytometer (BD Biosciences) and were analyzed using Flowjo software.

### Ki67 expression assay

Cells were fixed and permeabilized using the BD Cytofix/Cytoperm Fixation/Permeabilization Solution Kit (BD Biosciences) according to the manufacturer’s recommendations, followed by staining with anti Ki67-APC antibody (eBiosciences) or APC-conjugated IgG isotype control antibody (eBiosciences). Cell data were collected with a FACS Calibur flow cytometer (BD Biosciences) and were analyzed using Flowjo software.

### Luciferase reporter assay

The full-length 3′UTR of CCND1a and its short form and CCND1b 3′UTR were amplified from HEK293T genomic DNA. All 3′UTRs were cloned downstream of Renilla luciferase in the psiCHECK2 vector (Promega, Madison, Wisconsin, USA) following digestion with SalI and NotI. Cloning primers are listed in Supplementary Table 1.

For the luciferase reporter assay, 3.5 × 10e4 HEK293T cells per well were seeded into 48 well plates and transfected with the indicated plasmids (100 ng/well) and miRNA (100 nM) using Lipofectamine 3000 transfection reagent (Invitrogen) according to the manufacturer’s instructions. Cells were harvested 48 h after transfection, and relative luciferase units were quantified using Dual-Luciferase® Reporter Assays (Promega, Fitchburg, WI, USA) according to the manufacturer’s protocol on a GLOMAX 20/20 luminometer (Promega, Fitchburg, WI, USA). Three independent experiments were performed in triplicate. miRNAs are provided in Supplementary Table 1.

## Electronic supplementary material

supplementary data
