# Supplementary material for: Cell Cycle Regulation by Alternative Polyadenylation of CCND1
Source: Sci Rep. 2018 May 1;8:6824. doi: 10.1038/s41598-018-25141-0 (PMC5931507; doi:10.1038/s41598-018-25141-0)
Supplement: Supplementary file 1 — supplementary data [file 41598_2018_25141_MOESM1_ESM.docx]

**Cell Cycle Regulation by Alternative Polyadenylation of CCND1**

Qiong Wang^1^, Guopei He^1^, Mengmeng Hou^1^, Liutao Chen^1^, Shangwu Chen^1^, Anlong Xu^1,2 *^, Yonggui Fu^1*^

**^1^**State Key Laboratory for Biocontrol, Guangdong Province Key Laboratory of Pharmaceutical Functional Genes, Department of Biochemistry, School of Life Sciences, Sun Yat-sen University, Higher Education Mega Center, Guangzhou, 510006, P. R. China.

**^2^**Beijing University of Chinese Medicine, 11 Bei San Huan Dong Road, Chao-yang District, Beijing, 100029, P. R. China

^*^Corresponding authors:

Dr. Anlong Xu,

Department of Biochemistry, School of Life Sciences,

Sun Yat-sen University,

Higher Education Mega Center,

Guangzhou, 510006, China.

Tel.: +86 20 39332990 Fax: +86 20 39332950

Email: [lssxal@mail.sysu.edu.cn](mailto:lssxal@mail.sysu.edu.cn)

Dr. Yonggui Fu

Department of Biochemistry, School of Life Sciences,

Sun Yat-sen University,

Higher Education Mega Center,

Guangzhou, 510006, China.

Tel.: +86 20 39332958 Fax: +86 20 39332950

Email: [fuyg@mail.sysu.edu.cn](mailto:fuyg@mail.sysu.edu.cn)

**Supplementary Figures**


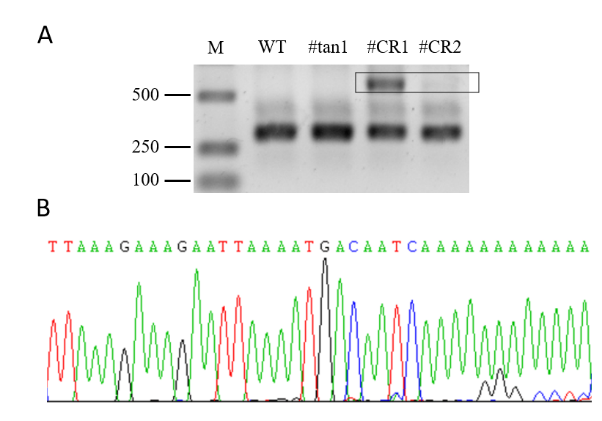


Supplementary Fig. 1. **Validation of the APA-1 site with 3'RACE.** A) Fragment of 530bp was amplified with 3'RACE method. B) Sanger sequencing validation.





Supplementary Fig. 2. **Validation of APA switching in the mutated cell lines.**

**
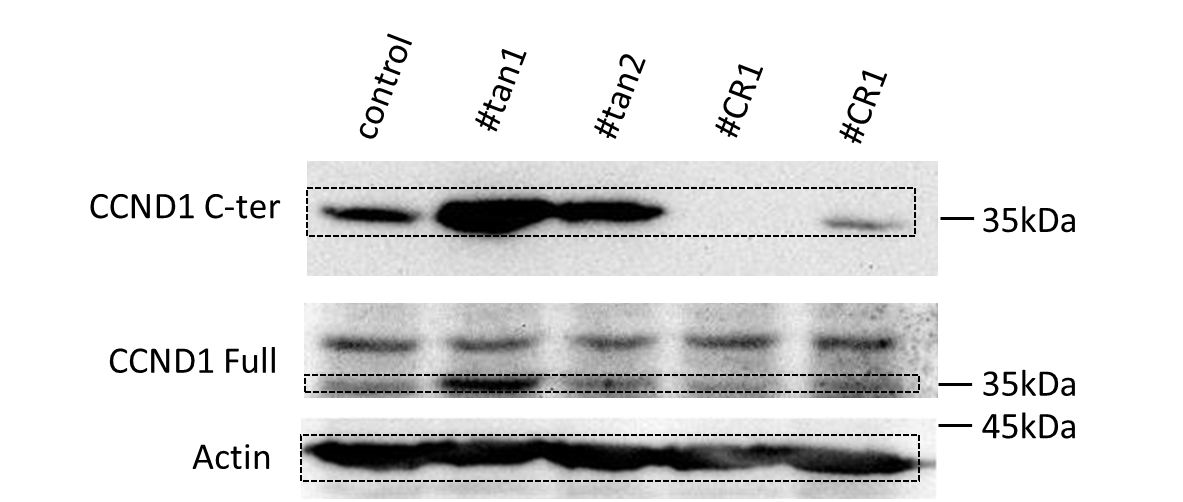
**

Supplementary Fig.3. **Full scan images of the westernblots.** In several cases, selected data were shown in the manuscript and the nitrocellulose membranes for westernblots were cut into strips to minimize the amount of antibodies that are necessary for analysis. Scans of entire nitrocellulose membranes strips are provided.

Supplemental Table 1. Primer and oligonucleotide sequences.

| sgRNA sequences | sgccnd1CR-1 | Sense: caccgCAAGTGTGACCCGGTAAGTG |
| --- | --- | --- |
|  |  | Anti-sense: aaacCACTTACCGGGTCACACTTGc |
|  | sgccnd1CR-2 | Sense: caccGGACATCACCCTCACTTACC |
|  |  | Anti-sense: aaacGGTAAGTGAGGGTGATGTCC |
|  | sgccnd1tan-1 | Sense: caccgATAAAAACGAGTTGATTATT |
|  |  | Anti-sense: aaacAATAATCAACTCGTTTTTATc |
|  | sgccnd1tan-2 | Sense: caccGGTTGTGCTACAGATGATAG |
|  |  | Anti-sense: aaacCTATCATCTGTAGCACAACC |
| ssODN sequences | ssODN870G/A | CCTACTACCGCCTCACACGCTTCCTCTCCAGAGTGATCAAGTGTGACCCAGTAAGTGAGGGTGATGTCCCAGGCAGCCTTGCCGGGGCTTACAGGGGGAG |
|  | ssODNtan | GGGGGAGGAGGGTTGTGCTACAGATGATAGAGGATTTTATACCCCAATAAAGGATCCTCAACTCGTTTTTATATTAATGTACTTGTTTCTCTGTTGTAAG |
| RFP-GFP reporter sequences | RPTCCND1CR-1 | RFP-GAA TTC CTC ACT TAC CGG GTC ACA CTT GCG ATC CAG **TGA**-GFP |
|  | RPTCCND1CR-2 | RFP-GAA TTC AGG ACA TCA CCC TCA CTT ACC GGG ATC CAG **TGA**-GFP |
|  | RPTCCND1tan-1 | RFP-GAA TTC CCA ATA ATC AAC TCG TTT TTA TAG ATC CAG **TGA**-GFP |
|  | RPTCCND1-tan-2 | RFP-GAA TTC CTC TAT CAT CTG TAG CAC AAC CAG ATC CAG **TGA**-GFP |
| PCR primers for RFLP | SurHDRCCND1-CR | Sense: TTTGTCATCGGCCAGAAATAGGA |
|  |  | Anti-sense: AGTTCTAGGAGCAGTGGAAGAAG |
|  | SurHDRCCND1-tan | Sense: CCTCCTCTCCGGAGCATTTTG |
|  |  | Anti-sense: ACCTTTTTCTTCTTGACTGGCAC |
| Primer for 3'RACE |  | ACCTAGTGCCACGGAAATGC |
| miRNA | hsa-miR-204-5p | uucccuuugucauccuaugccu |
|  | hsa-miR-193a3p | aacuggccuacaaagucccagu |
| Primers for 3′UTR clone | CCND1a-SUTR | Sense:  ATATCTCGAGGGGCGCCAGGCAGGCGGGC |
|  |  | Anit Sense: CGGAGCGGCCGCGGGGTATAAAATCCTCTATCA |
|  | CCND1a-LUTR | Sense: ATATCTCGAGGGGCGCCAGGCAGGCGGGC |
|  |  | Anti-sense: CCCGGCGGCCGCTCTAGACTTTCATGTTTGTCT |
|  | CCND1b-UTR | Sense: ATATCTCGAGCAAGGTTGGGGCTGGGGCTGG |
|  |  | Anti-sense: TATAGCGGCCGCGGCCACGCACATTGTGGC |
| Real-time PCR primers | CCND1-APA1-com | Sense: CGTCCATTGCGGAAGATCGTC |
|  |  | Anti-sense: TAGTTCATGGCCAGCGGGAA |
|  | CCND1-APA2-com | Sense: GGAAAGCTTCATTCTCCTTGTTG |
|  |  | Anti-sense: TTCTTTTGCTTAAGTCAGAGATGGAA |
|  | CCND1-APA2-extend | Sense: CTGTGACGCGCAAGTCTGAG |
|  |  | Anti-sense: TGCTGGAAACATGCCGGTTAC |
|  | CCND1-APA1-extend | Sense: GCCAATGGTCTGTGTGGTGAT |
|  |  | Anti-sense: ATTGGCCACGCACATTGTG |
